# Plasma Choline as a Diagnostic Biomarker in Slow Coronary Flow

**DOI:** 10.1155/2020/7361434

**Published:** 2020-01-25

**Authors:** Yuan-Ting Zhu, Ling-Ping Zhu, Zhen-Yu Wang, Xue-Ting Qiu, Wan-Zhou Wu, Wei-Wang Liu, Yu-Yu Feng, Wen-Kai Xiao, Xin Luo, Zhen-Yu Li, Chuan-Chang Li

**Affiliations:** ^1^Department of Geriatric Medicine, Xiangya Hospital, Central South University, Changsha 410008, China; ^2^Department of Cardiovascular Medicine, Xiangya Hospital, Central South University, Changsha 410008, China

## Abstract

**Aim:**

The slow coronary flow (SCF) phenomenon was characterized by delayed perfusion of epicardial arteries, and no obvious coronary artery lesion in coronary angiography. The prognosis of patients with slow coronary flow was poor. However, there is lack of rapid, simple, and accurate method for SCF diagnosis. This study aimed to explore the utility of plasma choline as a diagnostic biomarker for SCF.

**Methods:**

Patients with coronary artery stenosis <40% evaluated by the coronary angiogram method were recruited in this study and were grouped into normal coronary flow (NCF) and SCF by thrombolysis in myocardial infarction frame count (TFC). Plasma choline concentrations of patients with NCF and SCF were quantified by Ultra Performance Liquid Chromatography Tandem Mass Spectrometry. Correlation analysis was performed between plasma choline concentration and TFC. Receiver operating characteristic (ROC) curve analysis with or without confounding factor adjustment was applied to predict the diagnostic power of plasma choline in SCF.

**Results:**

Forty-four patients with SCF and 21 patients with NCF were included in this study. TFC in LAD, LCX, and RCA and mean TFC were significantly higher in patients with SCF in comparison with patients with NCF (32.67 ± 8.37 vs. 20.66 ± 3.41, *P*  <  0.01). Plasma choline level was obviously higher in patients with SCF when compared with patients with NCF (754.65 ± 238.18 vs. 635.79 ± 108.25, *P*=0.007). Plasma choline level had significantly positive correlation with Mean TFC (*r* = 0.364, *P*=0.002). Receiver operating characteristic (ROC) analysis showed that choline with or without confounding factor adjustment had an AUC score of 0.65 and 0.77, respectively.

**Conclusions:**

TFC were closely related with plasma choline level, and plasma choline can be a suitable and stable diagnostic biomarker for SCF.

## 1. Introduction

The slow coronary flow (SCF) phenomenon was an angiographic observation characterized by angiographically normal or near-normal coronary arteries with delayed opacification of the distal vasculature [1]. The SCF phenomenon was first proposed by Tambe et al. in 1972. Studies have found that the prevalence of SCF patients was about 1% of those who underwent coronary angiography. Previous researches have shown an increased risk of major adverse cardiovascular events (MACE) in patients with SCF, such as atherosclerosis [2], acute coronary syndrome [3], nonobstructive myocardial infarction [4], malignant arrhythmia [5], and metabolic syndrome, which was explained by vascular endothelial dysfunction, microangiopathy, inflammation, atherosclerosis, platelet activity and dysfunction, and insulin resistance [2, 6–10].

Choline, a saturated quaternary amine in all types of cells, participates in the lipid components of cell membranes [11]. Plasma choline level is considered to be related with several detrimental outcomes, including incidence of MACE and all-cause death, atherosclerosis, acute coronary syndrome, arrhythmia, and heart failure [12–16], and the main mechanism of choline-induced cardiovascular events is to promote a progressive inflammatory response which is attributed to aggregation of macrophages and CD36 receptors and enhance oxidative stress, endothelial dysfunction, and disorder lipid metabolism [17–19]. Therefore, SCF and choline-induced MACE may share a common underlying pathwayinflammation-related endothelial cell dysfunction during the individual pathological process.

Although patients with SCF often suffer a high morbidity and poor prognosis, it is generally regarded as a rare disease which can be ignored or overlooked easily. Moreover, coronary angiography is the gold standard for SCF diagnosis, but it is an expensive and invasive procedure with a certain degree of death, stroke, and myocardial infarction risk [20, 21]. Therefore, a rapid, simple, noninvasive, and accurate diagnostic method for SCF is highly desirable. Increasing number of studies has been providing crucial evidences that plasma choline can be employed as a stable and exact diagnostic indicator in the area of cancer, cardiac vascular disease, and acute ischemic stroke progression [22–24]. To our knowledge, the role of plasma choline in SCF has not been previously evaluated. In our study, we detected the plasma choline level of patients with normal coronary flow (NCF) and SCF and analyzed the relationship between choline and SCF to determine whether plasma choline can be served as a good diagnostic biomarker of SCF.

## 2. Methods

### 2.1. Study Population

A total of 1395 blood samples were randomly collected from patients who underwent coronary angiography between 2014 and 2017 in Xiangya Hospital. The inclusion criteria of our study were patients who were characterized by (1) having chest pain symptom or ischemic evidence on electrocardiogram, treadmill exercise test, or myocardial scintigraphy and (2) the detection of coronary angiography were normal (artery stenosis degree <40%) [25, 26]. The exclusion criteria of our study was confirmed coronary heart disease, coronary artery dissection, coronary artery spasm, obvious dilation of coronary artery, heart valves, cardiomyopathy, and cardiac insufficiency (ejection fraction <50%). Echocardiography showed left ventricular insufficiency and left ventricular hypertrophy, autoimmune diseases, tumors, systemic infectious diseases, renal insufficiency (serum creatinine >1.5 mg/dl), uncontrolled hypertension: systolic pressure >160 mmHg or diastolic pressure >105 mmHg, poor image quality of coronary angiography, patients with dysphagia, intestinal dysfunction, gastrointestinal surgery history, vitamin B supplementation, and antibiotics or probiotics in recent 6 months [26]. There were 65 patients included in our study based on inclusion and exclusion criteria. The study protocol was approved by the Ethics Committee of Xiangya Hospital, and informed consent was obtained from each patient.

### 2.2. Assessment of Thrombolysis in Myocardial Infarction (TIMI) Frame Count (TFC)

All patients underwent coronary angiography (CAG) with the standard Judkins method. Coronary flow rates were assessed by TFC, which was a classical method described by Gibson et al. [27], and standard images were obtained at 30 frames per second. The first frame used for TIMI frame counting is the frame in which the contrast agent enters both sides of the wall at the beginning of coronary artery and moves forward smoothly. The final frame is defined as the frame when the contrast agent enters the anatomical landmark of the distal vessels. The anatomical landmarks are defined as distal apical “eight-character” bifurcation in the left anterior descending (LAD), the furcation of the distal blunt margin branch in the left circumflex (LCX), and the first posterior branch of the left ventricle in the right coronary artery (RCA). Because the LAD is generally longer than LCX and RCA, the TIMI of LAD was divided by 1.7 to obtain corrected TIMI frame count (cTFC). The mean TIMI frame count for each subject was obtained by adding the corrected TFC of the LAD to the LCX and the RCA and then dividing the obtained value by 3 [27, 28].

### 2.3. Grouping Criteria for This Study

All patients in this study were grouped into NCF and SCF by the following criteria: patients with corrected TFC less than 27 frames per second in at least one of the LAD, LCX, and RCA were classified into the NCF group; patients with corrected TFC greater than 27 frames per second were diagnosed as SCF.

### 2.4. Laboratory Analysis

Blood samples were obtained from antecubital vein for analysis of biochemical and hematological data in the morning after an overnight fasting. For measurement of choline, TMAO, betaine, and L-carnitine, blood samples were obtained during coronary angiography and were centrifuged immediately with 3000 rpm for 10 minutes to obtain plasma samples. All samples were stored at −80°C until assayed. Plasma choline, TMAO, betaine, and L-carnitine were measured by ultra-high-performance liquid chromatography electrospray ionization mass spectrometry (RP-UHPLC-ESI-MS), and the internal standards choline-trimethyl-d9 (d9-choline), TMAO trimethyl-d9 (d9-TMAO), L-carnitine-trimethyl-d9 (d9- L-carnitine), and betaine-trimethyl-d9-methylene-d2 (d11-betaine) were added into plasma samples after protein precipitation, oscillated for 10 second, and centrifuged with 13200 rpm for 10 min, 200 *μ*l supernatant for detection by mass spectrometry system. The liquid phase system was ACQUITY UPLC I-Class (Waters, USA), and the mass spectrometry system was SCIEX 6500+ (SCIEX, USA).

### 2.5. Statistical Analysis

Continuous variables were expressed as mean ± standard error of the mean (SEM), and categorical variables were expressed as percent. Kolmogorov Smirnov test was used to evaluate the normal distribution of continuous variables. Student's *t*-test or Mann–Whitney *U*-test were used to compare the difference of continuous variables. The chi-square test was used to compare the difference of categorized variables. The correlations between plasma choline levels and mean TIMI frame count were assessed by the Pearson or Spearman correlation test. The diagnostic power of choline in SCF was assessed by receiver operating characteristic (ROC) curve. *P* value <0.05 was considered statistically significant. All statistical analyses were performed using SPSS for Windows version 11.5 (SPSS Inc., Chicago, Illinois, USA).

## 3. Results

### 3.1. Clinical Characteristics

A total of 65 subjects were recruited into our study, according to the grouping criteria, 21 of them were classified as patients with NCF (TFC < 27), and 44 patients were diagnosed as SCF (TFC < 27). The baseline demographic and clinical characteristics of the patients with SCF and NCF are presented in Table 1. There were no significant differences in the basic indexes of age, body mass index, heart rate, and systolic and diastolic blood pressure. In addition, there were also no obvious differences between patients with SCF and NCF in the terms of CAD risk factors, medication history, and basic biochemical indicators, such as serum triglyceride, total cholesterol, high-density lipoprotein cholesterol, low-density lipoprotein cholesterol, fasting glucose, creatinine, and high-sensitivity C-reactive protein. However, when compared with patients with NCF, patients with SCF showed significant predominance for male sex, alcohol consumption, and white blood cells (WBC) (*P*=0.026, *P*=0.035 and *P*=0.035, respectively).

### 3.2. Coronary Angiographic Findings between NCF and SCF Group

**Figure 1:**
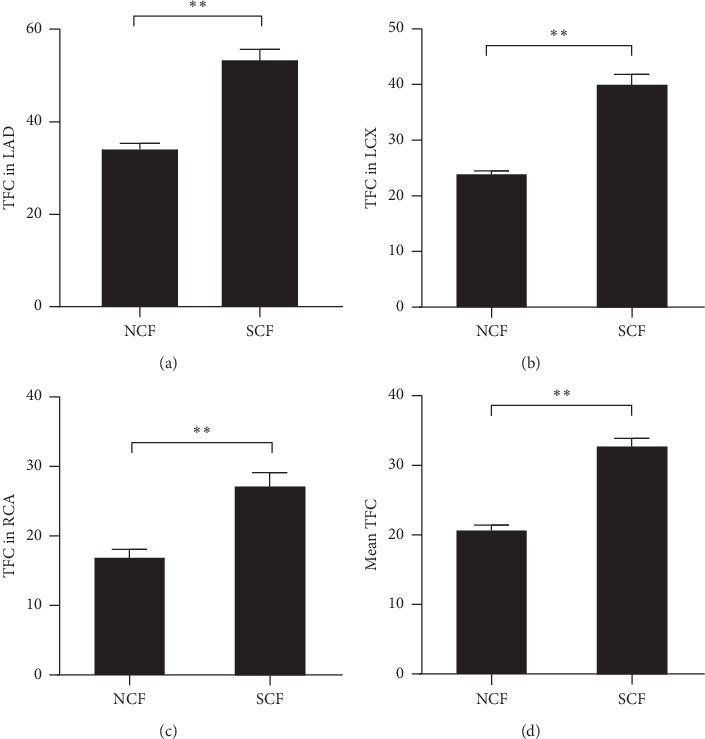
Thrombolysis in Myocardial Infarction (TIMI) frame count (TFC) in patients with NCF and SCF. (a) TFC in LAD between patients with NCF and SCF. (b) TFC in LCX between patients with NCF and SCF. (c) TFC in RCA between patients with NCF and SCF. (d) Mean TFC of LAD, LCX, and RCA between patients with NCF and SCF. LAD, left anterior descending; LCX, left circumflex; and RCA, right coronary artery. Data were expressed as mean ± SEM. ^*∗∗*^*P* < 0.01.

In order to evaluate TFC of all 3 epicardial coronary artery in patients with NCF and SCF. We assessed the TFC through coronary angiographic analysis of every patient. As shown in Figure 1, TFC of LAD, LCX, and RCA in patients with SCF was significantly higher compared with patients with NCF (53.88 ± 16.04 vs. 34.57 ± 5.90, *P* < 0.001, Figure 1(a); 39.95 ± 12.71 vs. 23.90 ± 2.71, *P* < 0.001, Figure 1(b); 32.67 ± 8.37 vs. 20.66 ± 3.41, *P* < 0.001, Figure 1(c), respectively). Furthermore, the mean of TFC in patients with SCF was also significantly higher than patients with NCF (32.67 ± 8.37 vs. 20.66 ± 3.41, *P* < 0.001, Figure 1(d)).

### 3.3. Plasma Choline Levels in NCF and SCF Group

**Figure 2:**
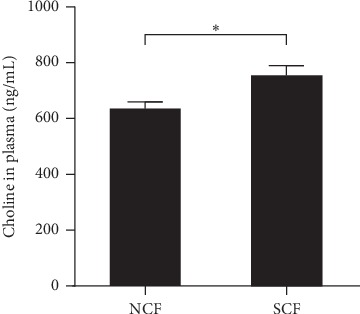
Plasma Choline levels in patients with NCF and SCF. Data were expressed as mean ± standard error of the mean.

To explore the role of choline in slow coronary flow, we performed RP-UHPLC-ESI-MS assay to determine the plasma choline concentration. As shown in Figure 2 plasma choline level was significantly higher in patients with SCF than those with NCF (754.65 ± 238.18 vs. 635.79 ± 108.25 mmol/L, *P*=0.007).

### 3.4. Associations between Plasma Choline and SCF

**Figure 3:**
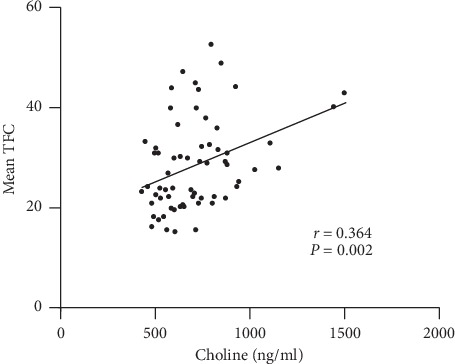
The relationship analysis between plasma choline level and mean TFC. *r*, Pearson correlation coefficient.

In order to further investigate the relationship between TFC value and plasma choline level, we applied the correlation analysis method for TFC value and plasma choline concentration in all subjects.  Figure 3 suggested that plasma choline level was positively correlated with mean TFC (*r* = 0.364, *P*=0.002).

### 3.5. Diagnostic Power of Choline in SCF

**Figure 4:**
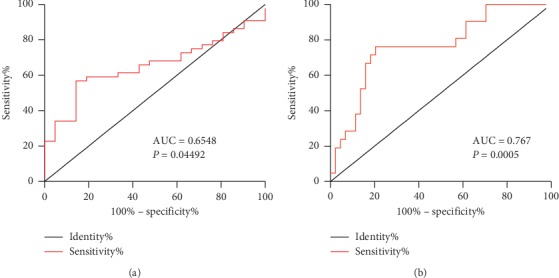
The diagnostic power of plasma choline in SCF. (a) Receiver operating characteristic (ROC) curve analysis of plasma choline level in patients with NCF and SCF. (b) Receiver operating characteristic (ROC) curve analysis of plasma choline level in patients with NCF and SCF after adjustment with sex, drinking, and white blood cell numbers. AUC, area under the curve.

We further performed receiver operating characteristic (ROC) analysis to determine whether choline can serve as an independent diagnostic indicator of SCF. As shown in Figure 4(a), the ROC analysis indicated a cutoff value of 673.5 ng/mL for plasma choline level to diagnose SCF with 56.8% sensitivity and 85.7% specificity, and the area under the ROC curve (AUC) was 0.6548 (95% CI: 0.523–0.786, *P*=0.044). In order to get rid of the effect of confounding factors in the diagnosis process, we adjusted the factors which increased significant differences between patients with NCF and SCF, including sex, drinking, and number of white blood cell. We found that diagnostic ability of plasma choline with 79.5% sensitivity and 76.2% specificity was significantly enhanced after adjusting with confounding factors (cut-off value, 1030 ng/mL; AUC, 0.767; 95% CI: 0.642–0.892 *P*=0.0005, Figure 4(b)).

### 3.6. Plasma Trimetylamine-Oxide, L-Carnitine, and Betaine in the NCF and SCF Group

**Figure 5:**
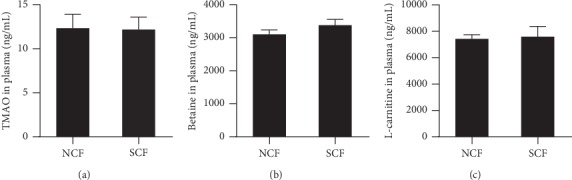
Plasma Trimetylamine-oxide (TMAO), L-carnitine, and betaine levels in the NCF group and SCF group. (a) Plasma TMAO concentration in patients with NCF and SCF, (b) plasma betaine level in patients with NCF and SCF, and (c) plasma L-carnitine level in patients with NCF and SCF. Data were expressed as mean ± standard error of the mean.

Choline, carnitine, betaine, and their metabolite Trimetylamine-oxide (TMAO) are closely related to adverse cardiovascular events, and we further studied the level of carnitine, betaine, and TMAO in plasma of patients with NCF and SCF by using RP-UHPLC-ESI-MS assay. The results showed that there was no significant difference in plasma TMAO, L-carnitine, and betaine between patients with NCF and SCF (Figure 5).

## 4. Discussion

As shown in the result, we concluded the following findings: (i) patients with SCF showed a significant predominance for male sex, alcohol consumption, and white blood cell numbers compared with patients with NCF; (ii) TFC of LAD, LCX, RCA, and mean TFC in patients with SCF were significantly higher compared with patients with NCF; (iii) the plasma choline level was obviously increased in the SCF group compared with the NCF group; (iv) the plasma choline level was positively correlated with mean TFC; and (v) plasma choline showed a significant diagnostic power in patients with SCF.

Prior studies have noted that inflammation is an important pathogenesis of SCF, and Li et al. showed that plasma c-reactive protein and interleukin-6 levels were significantly increased in the SCF group compared with the control group (all *P* < 0.01) [28]. Turhan et al. found that plasma ICAM-1, VCAM-1, and E-selectin levels were higher in the slow coronary flow group than in the control group (all *P* <  0.01), and there was a positive correlation between TFC and inflammatory mediators (*P* <  0.01) [29], indicating that patients with SCF might be characterized by vascular endothelial activation and response to inflammation. Coincidentally, in our study, we found that the number of white blood cells was significantly higher in the SCF group than in the NCF group, which was consistent with previous studies. However, multicenter and larger sample size will be needed to further verify the correlation between SCF and inflammation.

Choline is a water-soluble nutrient chemically defined as 2-hydroxyethyl-trimethyl ammonium hydroxide, which is an important element of B vitamins. Dietary choline mainly comes from red meat such as beef, pork, and eggs [30]. Several studies explored the role of choline in cardiovascular diseases, for example, Danne et al. proposed that whole blood choline level was related to cardiac death and nonfatal cardiac arrest [12], choline activated in leukocytes and platelets, secretion of matrixmetalloproteinases by macrophages, activation of macrophages by oxidized low-density lipoprotein, and represented a major feature of unstable or ruptured coronary plaque [31]. Zuo et al. proposed that plasma choline increased atrial fibrillation risk (AF), choline determined the methylation state of liver, and increased the susceptibility to AF [16, 32]. In our study, plasma choline was significantly upregulated in patients with SCF compared with patients with NCF, and a positive correlation between choline levels and TFC has been further confirmed. However, the underlying mechanism still remains uncertain.

It was reported that endothelial dysfunction was closely related to slow coronary flow [26]. Pohl and Busse indicated endothelium-derived NO is an important regulator of slow coronary flow with potent vasodilatory effects [33]. Ren et al. found that choline has been shown to decrease both the production and bioavailability endothelium-derived NO, increase ET-1 level, and accelerate oxidative stress. It reduces vascular compliance, accelerates vascular remodeling, increases vascular resistance, and limits blood flow [17]. Thus, choline may play an important role in regulating coronary flow. Wang et al. found choline can increase aortic macrophage and scavenger receptor CD36 content and the diffuse atherosclerotic may increase epicardial resistance and reduce coronary flow [19]. Besides, Jia et al. demonstrated that choline would dramatically increase the TNF-*α* and CRP level, promoting inflammatory response and inducing SCF [18]. These findings apparently support the concept that choline is closely related with SCF and elevated in patients with SCF.

Choline, as an organic compound, is an important component of the cell membrane, which is widely distributed, easily detected, and has good stability [34]. Previous studies showed plasma choline could serve as a stable and exact diagnostic indicator in the area of cancer. Yang et al. identified that choline may be a valuable potential diagnostic biomarker for neoplasm progression, recurrence, chemoradiotherapy, and prognosis [22]. Yu et al. verified plasma choline as a reasonable biomarker to diagnose the progression of acute ischemic stroke in terms of selectivity, linearity, sensitivity, precision, accuracy, carryover effect, and stability [35]. Besides, Danne et al. found whole blood choline can be a diagnostic biomarker of ACS in future clinical studies [12]. Although our study showed a similar biomarker role of plasma choline in SCF, there were some differences between our study and Danne's study: (1) there were different inclusion criteria, and compared with their 342 patients with ACS in the United States, we included 65 Chinese patients with acute or chronic chest pain and ischemia evidence; (2) Danne detected the plasma concentration in whole blood instead of plasma; and (3) their cut-off value was 28.2 *μ*mol/L, while our cut-off value was 1030 ng/ml. Plasma choline showed a significant diagnostic power with and without confounding adjustment. Therefore, plasma choline could be served as a reliable, convenient, and noninvasive diagnostic biomarker of SCF with a high specificity.

Choline metabolizes trimethylamine (TMA) by intestinal microorganisms, and then TMA is oxidized to TMAO by flavin-containing monooxygenase 3 (FMO3) in liver [36]. Trimethylamine-N-oxide (TMAO), as an intestinal metabolite of choline, plays an important role in mediating choline-induced cardiovascular disease [37–39]. TMAO is in turn derived from the diet red meat, L-carnitine or betaine, and some mechanisms connect between TMAO, choline, and cardiovascular disease [40–43]. Trøseid et al. observed that TMAO and choline levels were elevated in patients with chronic heart failure [44]. However, there was no significant difference of plasma TMAO and red meat-L-carnitine or betaine between patients with NCF and SCF in our study, and the following reasons could be considered: (1) the choline metabolite TMAO was affected by other precursors L-carnitine and betaine and (2) the individuals with differences of intestinal microorganisms and FMO3 activity.

## 5. Conclusion

In summary, the level of plasma choline was positively correlated with TFC and plasma choline can be a novel diagnostic biomarker for SCF.

## Figures and Tables

**Table 1:** Clinical characteristic of the study subject.

		NCF group (*n* = 21)	SCF group (*n* = 44)	*P* value
*Clinical and hemodynamic data*				
Age (years)		58 ± 10	56 ± 8	0.448
Male, *n* (%)		4 (19)	21 (47.7)	0.026
BMI (kg/m^2^)		24.03 ± 1.86	24.24 ± 3.09	0.785
Smoking, *n* (%)		4 (19)	17 (38.6)	0.114
Drinking, *n* (%)		2 (9.5)	15 (34.1)	0.035
SBP (mmHg)		128 ± 20	127 ± 16	0.883
DBP (mmHg)		75 ± 9	77 ± 10	0.425
HR (bpm)		70 ± 12	71 ± 11	0.650
Hypertension, *n* (%)		10 (47.6)	18 (40.9)	0.609
DM, *n* (%)		4 (19)	3 (6.8)	0.137
Hyperlipidemia, *n* (%)		3 (14.4)	14 (31.8)	0.133

*Baseline medication*				
ARB/ACEI, *n* (%)		10 (47.6)	24 (54.5)	0.601
BB, *n* (%)		11 (52.4)	32 (72.7)	0.105
CCB, *n* (%)		7 (33.3)	16 (36.4)	0.811
Nitrates, *n* (%)		4 (19)	12 (27.3)	0.472
Statins, *n* (%)		17 (81)	38 (86.4)	0.572
Antiplatelet agent, *n* (%)		17 (81)	40 (90.9)	0.253
Anticoagulant, *n* (%)		3 (14.3)	11 (25)	0.326

*Biochemical and hematological data*				
WBC (10^9^/L)		5.38 ± 1.63	6.16 ± 1.22	0.035
TG (mmol/L)		1.30 ± 0.62	1.68 ± 1.15	0.167
TC (mmol/L)		4.22 ± 0.69	4.16 ± 0.97	0.151
HDL (mmol/L)		1.43 ± 0.41	1.26 ± 0.26	0.087
LDL (mmol/L)		2.40 ± 0.65	2..42 ± 0.80	0.900
FPG (mmol/L)		5.41 ± 0.80	5.16 ± 0.84	0.261
Creatinine (mmol/L)		83.38 ± 30.19	79.94 ± 13.04	0.521
Hs-CRP (mg/L)		2.26 ± 2.30	2.30 ± 3.20	0.956

Data were expressed as mean ± standard error of the mean (SEM) or the number (%) of patients. NCF, normal coronary flow; SCF, slow coronary flow; BMI, body mass index; SBP, systolic blood pressure; DBP, diastolic blood pressure; HR, heart rate; DM, diabetes mellitus; ARB, angiotensin receptor blocker; ACEI, angiotensin converting enzyme inhibitor; BB, beta-blocker; CCB, calcium channel blocker; WBC, white blood cell; TG, triglyceride; TC, total cholesterol; HDL, high-density lipoprotein; LDL, low-density lipoprotein; FPG, fasting plasma glucose; Hs-CRP, high-sensitivity C-reactive protein;

## Data Availability

The datasets used and analyzed during the current study are available from the corresponding author on reasonable request.

## Additional Points

A multicenter and larger sample size study will be required in our future work.
